# Supplementary material for: Contact lens procurement and usage habits among adults in Sudan
Source: PLoS One. 2021 May 19;16(5):e0251987. doi: 10.1371/journal.pone.0251987 (PMC8133405; doi:10.1371/journal.pone.0251987)
Supplement: S1 Table — (DOCX) [file pone.0251987.s001.docx]

**Contact Lenses Usage and Procurement Habits in Sudan**

**This survey consists of questions related to contact lens usage and procurement habits and is designed for completion by adult contact lens wearers in Sudan. The survey should take about 5 minutes to complete. The survey is anonymous and your participation in this study is completely voluntary and you are free to withdraw at any time. Your completion of the survey implies your consent to participate in this research project.**

**If you have questions about this research, please contact:**

**Dr. Yazan Gammoh (**[**y.gammoh@ammanu.edu.jo**](mailto:y.gammoh@ammanu.edu.jo)**; gammohyazan@yahoo.com)**

**Patient number:** __________________________

**Participant demographics:**

Gender: Male / Female Age: years Smoker Yes/ No

**Occupation**:

Professional Management Clerical / Administrative Semi-skilled

Unskilled Retired Student Unemployed

**Educational Level**

None High school Undergraduate Postgraduate

**Refractive Error:**

None Myopia Hyperopia Myopic Astigmatism Hyperopic Astigmatism Keratoconus

| **Contact Lens Usage:** | | | | | | | | | | | |
| --- | --- | --- | --- | --- | --- | --- | --- | --- | --- | --- | --- |
| CL type | Soft spherical | | | | | Soft toric | | | Soft cosmetic | Soft multifocal | Rigid Gas permeable |
| CL Power (Spherical Equivalent) | > 5.00 Diopter | | | | | ≤5 Diopter | | | Plano |  | |
| CL Prescriber | Eye care practitioner | | | | | Friends and family | | | Self-prescribing |  | |
| CL Purchase place | Contact lens practitioner | | | | | Pharmacy | | | Internet |  | |
| CL Wearing experience (months) |  | | | | | | | | | | |
| Wearing time per day | 1-5 h | | | 6-11 h | | | ≥ 12 hours | | |  | |
| CL wearing modality (Recommended) | Daily disposable | | Monthly | | 3-6 months | | | Yearly | |  | |
| Overnight wearing (Recommended) | Yes | | | | | No | | | | Not mentioned | |
| Solution Type/Brand | Multi-purpose | | | | | Saline | | | | No solution | |
| Solution prescriber | Eye care practitioner | | | | | Pharmacist | | | | Not prescribed | |
| Contact lens case cleaning material | Tap water | Contact lens solution | | | | Saline | | | | Not applicable | |

| **Behaviors** | **Always** | **Frequently** | **Occasionally** | **Rarely** |
| --- | --- | --- | --- | --- |
| Overnight wearing |  |  |  |  |
| Sharing CL with others |  |  |  |  |
| Swimming with CL |  |  |  |  |
| Showering with CL |  |  |  |  |
| Using enough solution in lens case |  |  |  |  |
| Topping up solution |  |  |  |  |
| Solution bottle sharing |  |  |  |  |
| Checking the expiry date of solution |  |  |  |  |
| Hands washing before inserting CL |  |  |  |  |
| Hands washing before removal of CL |  |  |  |  |
| Rinsing lens with tap water |  |  |  |  |
| Rubbing, rinsing & soaking with prescribed solution |  |  |  |  |
| Cleaning lens case |  |  |  |  |
| Lens case replacement |  |  |  |  |
| Lens case sharing |  |  |  |  |
| Attending after-care visits |  |  |  |  |
