# Supplementary material for: Contact lens procurement and usage habits among adults in Sudan
Source: PLoS One. 2021 May 19;16(5):e0251987. doi: 10.1371/journal.pone.0251987 (PMC8133405; doi:10.1371/journal.pone.0251987)
Supplement: Attachment — Submitted filename: Response to Reviewers.doc [file pone.0251987.s004.doc]

**Re: PONE-D-21-03329
Contact lens procurement and usage habits among adults in Sudan**

**Dear Binaya Sapkota, PharmD
Academic Editor, PLOS ONE**

I would like to thank you and the esteemed reviewers for all the valuable comments and suggestions. Kindly find below a reply to the reviewers’ and editor’s comments. I sincerely hope it meets your requirements.

1. **Additional editor’s comments:**

| **Original comments** | **Reply by the author(s)** | **Changes done on section** |
| --- | --- | --- |
| Please assess the reporting of this observational study of clinical data using the STROBE checklist | Thank you for raising this important point. A sentence about STROBE statement has been included in the methods section.  Supplement S1 Table includes the STROBE checklist. | Lines 165-167  S1_Table file |
| Also, address the inherent limitations of observational studies including whether there are unsupported statements of causation; and whether the analysis was affected by confounding variables, a lack of generalizability, selective reporting, post hoc analyses and other. | I would like to thank you for binging my attention to this issue. The requested information has been included in the limitations section. | Limitations and recommendations section  Lines 431-442 |

2. **Reply to Reviewer 1’s comments:**

| **Original comments of the reviewer** | **Reply by the authors** | **Changes done on section** |
| --- | --- | --- |
| Line 100, 107 and 119: Replace semicolon with a comma | Thank you so much for raising my attention to this issue. The semicolon in each line has been changed to a comma. | Lines 98, 105, 117 |
| Line 122: delete one ‘in’ | One ‘in’ has been deleted. | Line 120 |
| Methods: How were the participants selected, was it random or everyone attending the clinics and hospitals? There might be a selection bias, which needs to be clarified, especially because around 92% participants were female. Was it because of selection method or is it a true representative of the population? | The following paragraph was added to the methods section: ‘All patients attending the clinics during the data collection period were invited to participate in the study. To further minimize selection bias, and to ensure full participation of patients, volunteer optometrists administered the questionnaire rather than the patients’ contact lens practitioners.’  The following statement was added in the limitations section: ‘In addition, the majority of contact lens wearers in the study were women, though this was expected as the majority of patients reporting to contact lens clinics are women, based on the experience of the authors.’ | Methods  Lines 154-157  Limitations and recommendations  Lines 431-442 |
| Line 222: Table 2- percent in CL power column doesn’t add to 100. | The percent has been edited and now add to 100 | Table 2 |
| Line 230: clean should be ‘cleaned’ | Clean has been changed to “cleaned” | Line 246 |
| Line 253: Please mention what statistical tests were used to derive these p values. | Mann-Whitney U-test and Kruskal-Wallis test were mentioned in table 4 title. | Table 4 title |
| Line 398: The statement ‘The majority of CL wearers in Sudan are females’ is not a correct statement. Authors did not collect the data to check this – this is not a population-based study but a cross-sectional study of a sample. Instead, they can write that a majority of CL wearers were female in the present study (not in Sudan). | The following statement was used instead:  ‘The majority of CL wearers in the current study were women’ | Conclusion  Line 412 |

3. **Reply to Reviewer 2’s comments:**

| **Original comments of the reviewer** | **Reply by the authors** | **Changes done on section** |
| --- | --- | --- |
| manuscript is not presented in an intelligible fashion and written in standard English. | Sections have been arranged as per the style requirement of the journal.  Subsections in what was previously the discussion section were removed, as the results and discussion section were merged as per the Journal’s style requirements.  Reporting of males and females as men and women when used as nouns have been corrected.  Spelling and grammar has been checked throughout the manuscript. | Throughout the manuscript |
| Deeper analysis can be done | STROBE statement checklist has been added.  Inherent limitations to observational studies have been addressed.  Type of statistical test used in table 4 was included. | Methods section  Limitations and recommendations section |
| Please make sure that you have followed the authors guidelines properly. | Authors’ guidelines and PLOS ONE’s style requirements have been followed | All manuscript and files |
| Please keep limitations and recommendation sections after conclusion section | Limitations and recommendations section is placed after the conclusion section | Lines 430-451 |

4. **Journal requirements:**

| **Journal requirement number** | **Journal requirement** | **Reply by the authors** | **Changes done on section** |
| --- | --- | --- | --- |
| 1 | Please ensure that your manuscript meets PLOS ONE's style requirements, including those for file naming. | Manuscript, tables, figures, and supplement files have been edited to meet PLOS ONE’s style requirements. | All files. |
| 2 | Please include additional information regarding the survey or questionnaire used in the study and ensure that you have provided sufficient details that others could replicate the analyses. | The questionnaire has been included as supplement S2 Table. | File S2_Table |
| 3 | Please change "female” or "male" to "woman” or "man" as appropriate, when used as a noun | All changes needed have been implemented | All manuscript |
| 4 | Your ethics statement should only appear in the Methods section of your manuscript. | The ethics statement has been only included in the methods section. | Methods section |

I would like to inform you that the changes that have been made based on the reviewer’s comments have enriched the manuscript, thank you for your consideration.

Yours sincerely,

Yazan Gammoh, PhD
