# Supplementary material for: Contact lens procurement and usage habits among adults in Sudan
Source: PLoS One. 2021 May 19;16(5):e0251987. doi: 10.1371/journal.pone.0251987 (PMC8133405; doi:10.1371/journal.pone.0251987)
Supplement: Attachment — Submitted filename: Response to Reviewers.doc [file pone.0251987.s006.doc]

**Re: PONE-D-21-03329
Contact lens procurement and usage habits among adults in Sudan**

**30 April 2021**

**Dear Binaya Sapkota, PharmD
Academic Editor, PLOS ONE**

I would like to thank you and the esteemed reviewers for all the valuable comments and suggestions. Allow me to thank Reviewer 1 for their encouraging words and the support expressed to publish data from developing countries with low-income like Sudan.

1. As both reviewers highlighted the need for English language editing, all the necessary changes are shown in the file “Revised Manuscript with Track Changes” to allow the esteemed reviewers to track all the changes. All the tracked changes have been incorporated into the manuscript text in the file “Manuscript”.

2. As the reviewers had specific comments regarding the inappropriate use of commas and semicolons, kindly find below the changes made specifically for the use of semicolons:

| **Line** | **Original text** | **Changes done** |
| --- | --- | --- |
| 47 | lenscase; | lenscase, |
| 56 | appointments; | appointments, |
| 83 | worldwide; | worldwide, |
| 98 | beauty salons and over the internet | beauty salons, and over the internet |
| 122 | infrequent cleaning and discarding of the lens case; improper use of lens care products; use of tap | infrequent cleaning and discarding of the lens case, improper use of lens care products, use of tap |
| 125 | compliance to CL wear and care; age, gender and level of education are risk factors | compliance to CL wear and care, age, gender, and level of education are risk factors |
| 132 | CL wearers such as daily disposable (DD) CL wearers [21]; females [7]; or students | CL wearers such as daily disposable (DD) CL wearers [21], females [7], or students |
| 233 | Adult CL wearers; 18 years of age and above, | Adult CL wearers 18 years of age and above, |
| 269 | Participants’ demographics including, age; gender; occupation; level of education, type of | Participants’ demographics including age, gender, occupation, level of education, type of |
| 272 | CL wear profile including CL power and type; CL modality and replacement schedule; source | CL wear profile including CL power and type, CL modality and replacement schedule, source |
| 275 | to participants when they were not able to recall the product in question; a strategy that has | to participants when they were not able to recall the product in question, a strategy that has |
| 292 | CL usage behavior including overnight wear; CL sharing; swimming and showering | CL usage behavior including overnight wear, CL sharing, swimming and showering |
| 296 | with scores 1 to 4 assigned to responses ranging from “Always” to “Rarely”; | with scores 1 to 4 assigned to responses ranging from “Always” to “Rarely”, |
| 298 | ranging from “Rarely” to “Always”; respectively. | ranging from “Rarely” to “Always”, respectively. |
| 299 | before insertion and removal of CL; and | before insertion and removal of CL, and |
| 304 | “Always” to “Rarely”; sharing lens case | “Always” to “Rarely”, sharing lens case |
| 351-2 | (range; 18 to 45 years). | (range: 18 to 45 years). |
| 400 | Based on the compliance rates observed in Fig1; contact lens behaviors | Based on the compliance rates observed in Fig1, CL behaviors |
| 405-7 | showering with CL. Moderate compliance level observed for sharing solution bottle; handwashing before CL removal; topping up solution; checking the expiry date of the solution; lens case replacement and cleaning lens case. | showering with CL. Moderate compliance levels were observed for sharing solution bottle, handwashing before CL removal, topping up solution, checking the expiry date of the solution, lens case replacement, and cleaning lens case. |
| 410 | A group of factors; mainly gender, | A group of factors, mainly gender, |
| 432 | CL wear and care; including levels of | CL wear and care, including levels of |
| 512 | struggling with trachoma; a disease | struggling with trachoma. Trachoma is a disease |
| 518 | at the university hospital; where proper CL | at the university hospital, where proper CL |
| 551 | any of the study’s participants; due to the lack of access to | any of the study’s participants, due to the lack of access to |
| 582 | beauty salons [7]; thus, it is | beauty salons [7]. Thus, it is |
| 646 | of the level of compliance; due to the expected reluctance | of the level of compliance, due to the expected reluctance |

Yours sincerely,

Yazan Gammoh, PhD
